# Theranostic Verteporfin-Conjugated Upconversion Nanoparticles for Cancer Treatment

**DOI:** 10.3390/nano15221690

**Published:** 2025-11-07

**Authors:** Oleksandr Shapoval, Vitalii Patsula, David Větvička, Miroslav Šlouf, Martina Kabešová, Taras Vasylyshyn, Ludmila Maffei Svobodová, Magdalena Konefal, Olga Kočková, Jan Pankrác, Petr Matouš, Vít Herynek, Daniel Horák

**Affiliations:** 1 Institute of Macromolecular Chemistry, Czech Academy of Sciences, Heyrovského nám. 2, 162 06 Prague, Czech Republic; patsula@imc.cas.cz (V.P.); slouf@imc.cas.cz (M.Š.); vasylyshyn@imc.cas.cz (T.V.); kockova@imc.cas.cz (O.K.); 2 Institute of Biophysics and Informatics, First Faculty of Medicine, Charles University, Salmovská 1, 120 00 Prague, Czech Republic; david.vetvicka@lf1.cuni.cz (D.V.); martina.kabesova@lf1.cuni.cz (M.K.); ludmila.maffei@lf1.cuni.cz (L.M.S.); 3 NanoBioMedical Centre, Adam Mickiewicz University, Wszechnicy Piastowskiej 3, 61-614 Poznań, Poland; magdalena.konefal@amu.edu.pl; 4 Center for Advanced Preclinical Imaging, First Faculty of Medicine, Charles University, Salmovská 3, 120 00 Prague, Czech Republicpetr.matous@lf1.cuni.cz (P.M.); vit.herynek@lf1.cuni.cz (V.H.)

**Keywords:** nanoparticles, verteporfin, photodynamic therapy, pancreatic cancer, imaging

## Abstract

Photodynamic therapy (PDT) is a highly selective, clinically approved, minimally invasive technique that effectively eliminates cancer cells. Its effectiveness is limited by poor light penetration into tissue and the hydrophobic nature of photosensitizers, highlighting the need for new approaches to treatment. Here, a theranostic upconversion nanoplatform, consisting of a NaYF_4_:Yb,Er,Tm,Fe core and a NaHoF_4_ shell codoped with Yb, Nd, Gd and Tb ions, was designed to enhance PDT outcomes by integrating multi-wavelength upconversion luminescence, *T*_2_-weighted magnetic resonance imaging (MRI) and PDT. The synthesized core–shell upconversion nanoparticles (CS-UCNPs) were coated with new verteporfin (VP)-conjugated alendronate-terminated poly(*N*,*N*-dimethylacrylamide-*co*-2-aminoethyl acrylate) [Ale-P(DMA-AEA)] grafted with poly(ethylene glycol) (PEG). Under 980 nm NIR irradiation, CS-UCNP@Ale-P(DMA-AEA)-PEG-VP nanoparticles generated reactive oxygen species (ROS) due to the efficient energy transfer between CS-UCNPs and VP. In a pilot preclinical study, intratumoral administration of nanoparticle conjugates to mice, followed by exposure to NIR light, induced necrosis of pancreatic tumor and suppressed its growth.

## 1. Introduction

Cancer remains a major threat to human health, causing millions of deaths each year [1]. Although surgery, chemo-, and radiation therapies are commonly used therapeutic methods, their effectiveness is often unsatisfactory due to low patient response [2]. This highlights the importance of developing new effective treatments that will reduce overall cancer mortality.

Photodynamic therapy (PDT) is a clinically applicable approach designed as a promising alternative for treating unresectable or therapy-resistant tumors. PDT utilizes non-toxic light-activated photosensitizers (PSs) to generate reactive oxygen species (ROS), which effectively target and destroy tumor cells [3,4]. The growing popularity of PDT is explained by its advantages, including precise controllability, minimal drug resistance and reduced long-term side effects. ROS, such as singlet oxygen (^1^O_2_), hydrogen peroxide (H_2_O_2_), superoxide radicals (O_2_^•−^) and hydroxyl radicals (∙OH), trigger biological processes that directly kill cells, induce inflammation and disrupt tumor vasculature. However, the short diffusion distance of ROS within cells and tissues (<50 nm) requires PS to be localized in close proximity to a target tumor. Most PSs, typically hydrophobic porphyrins with large heteroaromatic rings, tend to aggregate in aqueous biological environments due to π–π stacking and hydrophobic interactions. This leads to fluorescence quenching and reduced ROS generation efficiency [5]. Additionally, the reliance on visible or ultraviolet light for PS excitation limits penetration depth due to absorption and scattering by biological tissues, rendering PDT ineffective for internal or large tumors [6]. In order to realize the full potential of PDT in cancer treatment, it is crucial to develop efficient delivery platforms, overcoming the above obstacles.

New nanomaterials engineered with the required functional properties allow the development of highly selective PDT platforms that integrate imaging and therapy. In particular, upconversion nanoparticles (UCNPs), which emit visible light under near-infrared (NIR) excitation, have emerged as a powerful tool to enhance PDT by transferring energy to PS [7,8]. NIR light takes advantage of the biological “optical transparency window” (700–1000 nm), allowing deeper tissue penetration with reduced scattering and minimal autofluorescence interference [9]. Conjugating PS with UCNPs also enhances the solubility and colloidal stability of hydrophobic PS, preventing its aggregation, avoiding self-quenching and increasing ROS yield [10]. Several strategies, including physical adsorption, encapsulation and covalent conjugation, have been developed to construct water-dispersible PS–UCNP complexes [11,12]. Direct physical adsorption of PS to UCNP surface via hydrophobic interactions or van der Waals bonding, using porous silica [13], polymers [14] and crosslinked or PEGylated lipids [15,16], offers a straightforward approach to control PS loading, but is prone to PS leaching [17]. Encapsulation enhances PDT specificity and efficacy but is limited by low energy conversion and poor PS loading, which reduces overall PDT effectiveness and limits its use in tumor treatments in vivo [18]. In contrast, covalent conjugation of PS to a polymer on UCNP surface prevents PS leakage, enhances loading efficiency and improves PDT outcomes [19]. For this purpose, Rose Bengal-poly(ethylene glycol)- [20], Rose Bengal-silica- [21], zinc phthalocyanine-poly(allylamine)- [22], chlorin-e6-polyethylenimine- [23], hematoporphyrin-silica- [24], protoporphyrin IX-poly(ethylene glycol)- [25], and chlorin e6-pullulan-conjugated UCNPs [7] demonstrated enhanced NIR-activated PDT. As a result, UCNP-mediated PDT enables effective treatment of deeper tumors compared to conventional visible-light PDT.

The doping of UCNPs with various lanthanide and transition metal ions further enhances their utility in theranostic (therapeutic and diagnostic) applications by modulating upconversion luminescence [26,27]. They exhibit unique optical properties such as high-efficient emission in the visible and infrared range, long excited-state lifetimes and narrow absorption/emission bands [28]. These features provide a high signal-to-noise ratio and resist photobleaching, making UCNPs ideal for bioimaging and light-based therapy. Strategic selections of dopant ions and adoption of a core–shell architecture enable the integration of multiple modalities into a single UCNP platform. This integration is becoming attractive for image-guided PDT, tumor resection and post-surgery evaluation, where the precise visualization of malignant tissues is fundamental to ensuring the treatment and complete resection of the tumor [29]. For instance, incorporating Ho^3+^, Yb^3+^, Tm^3+^ and Er^3+^ ions into a single nanocrystal not only greatly promotes the upconversion luminescence intensity and efficiency but also endows it with imaging capabilities along with PDT activation [30]. At the same time, doping with Yb^3+^, Gd^3+^, Ho^3+^ and Tb^3+^ ions imparts magnetic properties suitable for magnetic resonance imaging (MRI) [31]. Due to their short electronic relaxation time and large effective magnetic moment (µ_eff_ = 7.9–10.6 μB), such particles are promising *T*_1_ or *T*_2_ (*T*_2_*) MRI contrast agents. While paramagnetic Gd^3+^ ions provide predominant *T*_1_ contrast in clinical MRI, other lanthanide ions generate strong negative contrast in *T*_2_-weighted MRI, enabling sensitive in vivo imaging with simultaneous luminescence-based optical tracking [30,32]. The core–shell design protects the core from luminescence quenching caused by the surrounding media, while the outer shell is doped with lanthanide ions, enhancing MRI signal and shortening relaxation times. Utilizing the optical transparency of infrared light and the multifunctionality of lanthanide dopants, the nanomaterials can probe deeper pathological areas, provide real-time diagnostic imaging (both optical and magnetic) and deliver highly precise PDT against cancerous tissues.

**Scheme 1:**
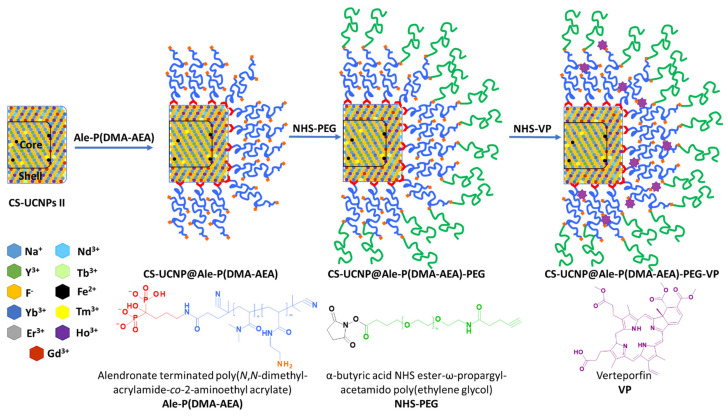
Schematic representation of the synthetic procedures for the preparation of CS-UCNP@Ale-P(DMA-AEA)-PEG-VP nanoparticles.

Herein, we designed and synthesized surface-engineered upconversion NaYF_4_:Yb,Er,Tm,Fe@NaHoF_4_ nanoparticles with covalently conjugated verteporfin (VP) for PDT and bimodal imaging (Scheme 1). Additional codoping of CS-UCNPs with Yb^3+^, Gd^3+^, Nd^3+^, and Tb^3+^ ions provided upconversion luminescence and *T*_2_-weighted MRI contrast. Coating CS-UCNPs with new VP-conjugated alendronate-terminated poly(*N,N*-dimethylacrylamide-*co*-2-aminoethylacryl acrylate)-*graft*-poly(ethylene glycol) [P(DMA-AEA)-PEG-Ale] ensured excellent colloidal stability in physiological fluids and efficient ROS generation under 980 nm irradiation. The photodynamic activity and toxicity of nanoparticles were examined in vitro and in vivo in a pilot therapeutic experiment.

## 2. Experimental Section

### 2.1. Materials

*N*-Hydroxysuccinimide (NHS; 98%), *N*,*N′*-dicyclohexylcarbodiimide (DCC; 99%), ammonium fluoride (NH_4_F; 99.9%), chlorides of yttrium (YCl_3_; 99%), erbium (ErCl_3_∙6H_2_O; 99%), ytterbium (YbCl_3_; 99%), thulium (TmCl_3_; 99.9%), terbium (TbCl_3_; 99.9%), neodymium (NdCl_3_; 99.9%), gadolinium (GdCl_3_; 99.9%), holmium (HoCl_3_; 99.9%), and iron (FeCl_2_∙4H_2_O), octadec-1-ene (90%), *N*,*N*-diisopropylethylamine (DIPEA; ≥99%), ethylenediaminetetraacetic acid (EDTA), phosphate-buffered saline (PBS; pH 7.4), Dulbecco’s modified Eagle medium (DMEM), fetal bovine serum (FBS), trypsin, 1,3-diphenylisobenzofuran (DPBF; 97%), phenazine methosulfate (PMS), and sodium 3′-[1-(phenylaminocarbonyl)-3,4-tetrazolium]-bis(4-methoxy-6-nitro)benzenesulfonic acid hydrate (XTT) were purchased from Merck (Darmstadt, Germany). *N*,*N*-Dimethylformamide (DMF; 99.8%) was obtained from Iris Biotech (Marktredwitz, Germany). Hexane (99.9%), ethanol (99.8%), hydrochloric acid (35%), methanol (99.9%), ethyl acetate (99.9%), oleic acid, sodium chloride and sodium hydroxide were from Lach-Ner (Neratovice, Czech Republic). Verteporfin (VP; Visudyne^®^; Scheme 1) was provided by MedChemExpress (Monmouth Junction, NJ, USA). α-Butyric acid NHS ester-ω-propargylacetamido poly(ethylene glycol) (NHS-PEG; *M*_n_ = 5000 g/mol; Scheme 1) was purchased from Rapp Polymere (Tuebingen, Germany). The remaining chemicals were purchased from commercial sources and utilized without additional purification. Alendronate-terminated poly(*N*,*N*-dimethylacrylamide-*co*-2-aminoethyl acrylate) [Ale-P(DMA-AEA)] containing 10 mol.% of 2-aminoethyl acrylate (*M*_n_ = 11 kg/mol; *M*_w_/*M*_n_ = 1.2; Scheme 1) was synthesized according to the literature [33]. Buffers and solutions were prepared from ultrapure demineralized water (conductivity <0.1 μS/cm) obtained by reverse osmosis with UV treatment (Milli-Q Gradient A10 system; Millipore; Molsheim, France). This distilled demineralized water was used throughout the experimental work.

### 2.2. Synthesis of Core and Core–Shell Nanoparticles

Core NaYF_4_:Yb,Er,Tm,Fe nanoparticles were prepared according to a previously described procedure, with slight modifications [34]. A mixture of YCl_3_ (72.5 or 77.5 mol.%), YbCl_3_ (20 mol.%), ErCl_3_∙6H_2_O (5 mol.%), TmCl_3_ (0.5 mol.%), and FeCl_2_∙4H_2_O (0 or 5 mol.%) was charged in a 100-mL three-neck flask with oleic acid (6 mL) and octadec-1-ene (15 mL). The flask was heated to 170 °C for 30 min with stirring under an argon atmosphere. After cooling to room temperature (RT), a solution of NaOH and NH_4_F (2.5/4 mol/mol) in methanol (20 mL) was added dropwise. The mixture was gently heated to 120 °C for 1.5 h under an argon atmosphere until methanol evaporated. The temperature was then increased to 300 °C for 1.5 h and the mixture was cooled to RT. The resulting NaYF_4_:Yb,Er,Tm (C-UCNPs I) or NaYF_4_:Yb,Er,Tm,Fe nanoparticles (C-UCNPs II) were washed with hexane/ethanol mixture (1/4 *v/v*) four times, collected by centrifugation (3460 rcf) for 30 min and redispersed in hexane.

Core–shell NaYF_4_:Yb,Er,Tm,Fe@NaHoF_4_ nanoparticles codoped with Gd,Yb,Nd, and Tb were synthesized according to an earlier described procedure [35]. First, 0.6 mmol of lanthanide chlorides (60 or 100 mol.% Ho^3+^, 0 or 10 mol.% Gd^3+^, 0 or 10 mol.% Yb^3+^, 0 or 10 mol.% Nd^3+^, and 0 or 10 mol.% Tb^3+^) were dissolved in octadec-1-ene (9 mL) and oleic acid (6 mL), the solution was heated at 160 °C for 30 min with magnetic stirring under an argon atmosphere and then cooled to RT. A methanolic solution of NaOH and NH_4_F (2.5/4 mol/mol) was separately mixed with 15 mL of hexane dispersion of C-UCNPs II nanoparticles (150 mg) and added dropwise into the reaction mixture and the temperature was increased to 70 °C to evaporate methanol and hexane. Heating was continued at 300 °C for 1.5 h and the mixture was subsequently cooled to RT under an argon flow. The resulting NaYF_4_:Yb,Er,Fe@NaHoF_4_ (CS-UCNPs I) or NaYF_4_:Yb,Er,Fe@NaHoF_4_: Gd,Yb,Nd,Tb core–shell nanoparticles (CS-UCNPs II) were collected by centrifugation (3460 rcf) for 30 min and washed with hexane/ethanol, ethanol, ethanol/water, and finally water using sedimentation and redispersion.

### 2.3. Modification of CS-UCNPs with Ale-P(DMA-AEA) and Grafting with Poly(ethylene glycol)

The dispersion of CS-UCNPs II (50 mg) in water (2.85 mL) was added to an aqueous solution of Ale-P(DMA-AEA) (50 mg; 1.65 mL) under sonication (Ultrasonic Homogenizer UP200S Hielscher; 20% power). After 1 min of sonication, the reaction mixture was stirred at 80 °C for 19 h. Resulting CS-UCNP@Ale-P(DMA-AEA) nanoparticles were separated by centrifugation (15,000 rcf) for 45 min, twice washed with water (4 mL) and redispersed in 1 mL of water. To the obtained nanoparticle dispersion, ethanol (1 mL) and DIPEA (40 µL) were added, and the mixture was stirred for 20 min. After that, the particles were separated by centrifugation (15,000 rcf) for 30 min, washed with water (2 mL) and the dispersion was centrifuged again. For the PEGylation, CS-UCNP@Ale-P(DMA-AEA) nanoparticles were redispersed in DMF (1 mL) and mixed with solution of NHS-PEG (33 mg) in DMF (1 mL) for 17 h under stirring (900 rpm). The resulting CS-UCNP@Ale-P(DMA-AEA)-PEG nanoparticles were collected by centrifugation (15,000 rcf) for 45 min, washed with DMSO (2 mL), and redispersed in it.

### 2.4. Conjugation of VP to CS-UCNP@Ale-P(DMA-AEA)-PEG Nanoparticles

The conjugation of VP to the nanoparticles was initiated by its activation with *N*-hydroxysuccinimide (NHS) through carbodiimide chemistry. DCC (0.59 mg; 2.84 µmol) and NHS (0.34 mg; 2.92 µmol) were briefly added to a solution of VP (2 mg; 2.78 µmol) in DMSO (1 mL), and the mixture was stirred at RT for 18 h in the dark. Then, the solution of NHS-activated VP was filtered through a 0.2 µm polyvinylidene fluoride membrane and stored in the fridge in the dark. The dispersion of CS-UCNP@Ale-P(DMA-AEA)-PEG nanoparticles (15 mg) in DMSO (0.5 mL) was added to the solution of NHS-activated VP (0.4 mg) in DMSO (0.2 mL) and stirred for 48 h in the dark. The resulting CS-UCNP@Ale-P(DMA-AEA)-PEG-VP nanoparticles were separated by centrifugation (15,000 rcf) for 45 min, washed twice with ethanol (1 mL) and sterile water (1 mL) and redispersed in it.

### 2.5. Characterization of Nanoparticles

The particles were monitored by a TECNAI Spirit G_2_ transmission electron microscope (TEM; FEI; Brno, Czech Republic) equipped with a detector for energy dispersive spectroscopy (EDAX; Mahwah, NJ, USA). Each sample was visualized with bright field imaging (TEM/BF), its elemental composition was verified with energy dispersive spectroscopy (TEM/EDS), and the crystalline structure was identified by selected area electron diffraction (TEM/SAED) using the freeware program package EDIFF [36]. TEM/BF micrographs were used for the determination of the number-average diameter (*D*_n_) of particles and dispersity (*Ð*) by analyzing at least 200 objects from micrographs as described elsewhere [37]. Dynamic light scattering (DLS) was measured using a Zetasizer Ultra instrument (Malvern Instruments; Malvern, UK) to determine the hydrodynamic diameter *D*_h_, polydispersity *PD* and ζ-potential of particles. The excitation and emission luminescence spectra were measured using an FS5 spectrofluorometer (Edinburgh Instruments; Edinburgh, UK) equipped with pulsed and continuous (150 W) xenon lamps and MDL-III-980 infrared diode laser with a nominal power of 2 W (beam size 5 × 8 mm^2^). X-ray diffraction (XRD) patterns of particles were measured with CuKα radiation (λ = 1.54 Å) using a high-resolution GNR Explorer diffractometer (Novara, Italy) with a Mythen 1K strip detector (STOE; Darmstadt, Germany) with Bragg–Brentano geometry, 10–80° 2*Ө* range, 0.1° step and 15 s time of each step. Lanthanum hexaboride SRM–660a NIST standard (US National Institute of Standards and Technology; Gaithersburg, MD, USA) [38] was used to determine the instrumental resolution. The crystallite sizes were estimated according to the Scherrer Equation (1):

*D*_XRD_ = K·*λ*/βcos*Ө*, (1)

where *D*_XRD_ is crystallite size, K = 0.89 is the Scherrer constant, λ = 1.54 Å is the X-ray wavelength, β is a full width at peak half-maximum, and *Ө* is the Bragg angle [39]. The degree of crystallinity was calculated as the proportion of the crystalline part relative to the sum of the amorphous and the crystalline parts of the spectra. FTIR spectra were obtained with a 100T FTIR spectrometer (Perkin-Elmer; Waltham, MA, USA) equipped with a Specac MKII Golden Gate single attenuated total reflection (ATR). Thermogravimetric analysis (TGA) of particles was conducted in air with a Perkin Elmer TGA 7 analyzer (Norwalk, CT, USA) over the temperature range 30–700 °C at a constant heating rate of 10 °C/min. Samples for elemental analysis (2–10 mg) were digested with HNO_3_ (0.3 mL) and HClO_4_ (1 mL) in a Biotage Initiator microwave reactor (Uppsala, Sweden). After the digestion, the concentrations of lanthanides and iron in the particles were determined with NexION 2000B inductively coupled plasma mass (ICP-MS) and Perkin Elmer 3110 atomic absorption spectrometers (AAS; Waltham, MA, USA), respectively. The content of VP in the nanoparticles was determined by a Specord 250 Plus UV–Vis spectrophotometer (Analytik Jena; Jena, Germany) at 690 nm using the established molar extinction coefficient 34,895 M^−1^cm^−1^ in DMSO/water (9/0.5 *v/v*) [40]. Singlet oxygen generation was monitored using a DPBF probe with a Specord 250 Plus UV–Vis spectrophotometer at 350–650 nm as a function of exposure time after irradiation with 980 nm laser or a continuous 150 W xenon lamp at 700 nm, according to previously described methodology [34].

### 2.6. Magnetic Resonance Relaxometry and Imaging

*T*_1_ and *T*_2_ relaxation times of aqueous dispersions of C- and CS-UCNPs at different concentrations were measured on a 0.5 T Bruker Minispec relaxometer (Bruker BioSpin; Ettlingen, Germany) at 23 and 37 °C. The relaxivities *r*_1_ and *r*_2_ were calculated using least-squares curve fitting of *R*_1_ and *R*_2_ relaxation rates (s^−1^) versus metal ion concentrations (mmol/L) after the contribution of water was deducted. MRI of CS-UCNPs II phantoms in water was performed on a preclinical 7 T scanner (MR Solutions; Guildford, UK) equipped with a mouse body volume coil. *T*_2_*-weighted gradient echo sequence (flip angle 90°, echo spacing TE = 5 ms, repetition time TR = 1000 ms) was used with slice thickness 2 mm, field of view 30 × 60 mm^2^ and matrix 128 × 256, and the same geometry.

### 2.7. XTT Cell Viability Assay

The XTT test was performed on 96-well plates (TPP Techno Plastic Products; Trasadingen, Switzerland; 3 × 10^4^ cells; 200 μL per well) according to the manufacturer’s protocol. The PaTu-8902 pancreatic adenocarcinoma cell line (Leibniz Institute; Berlin, Germany) was cultured at 37 °C in a 5% CO_2_ atmosphere in DMEM supplemented with glucose (4.5 g/L), 1 mM sodium pyruvate, 1% penicillin/streptomycin (Gibco; Waltham, MA, USA) and 10% FBS. The cell suspensions were incubated in 0.025% trypsin and 0.01% EDTA in PBS for 5–10 min to achieve detachment, resuspended in growth medium and seeded into plates. The next day, the dispersions of Ale-P(DMA-AEA)-PEG-coated CS-UCNPs II nanoparticles with or without conjugated VP (50 μL; 0.05–300 µg/mL) were added and the plates were incubated for 48 h in a CO_2_ incubator. After removing the supernatant, 25 μL of a mixture containing XTT and PMS was added to the plates and incubation continued for 2 h. The absorbance was measured on a Tecan Infinite^®^ F50 plate reader (Schoeller; Prague, Czech Republic) at 450 nm with a reference wavelength of 620 nm.

### 2.8. Hemolysis Assay

Blood was collected retro-orbitally from mice into potassium oxalate-containing tubes and then centrifuged in three steps at 3000× *g* for 5 min each. The collected red blood cells (RBCs) were resuspended in PBS at a dilution ratio of 1:50 (*v/v*). For hemolysis evaluation, the dispersions of Ale-P(DMA-AEA)-PEG-coated CS-UCNPs II nanoparticles with or without conjugated VP were mixed with the RBC suspension in a 96-well U-bottom plate. Samples were incubated for 1 h at 37 °C in the dark and at RT in the presence of light and the plate was centrifuged again at 3000× *g* for 5 min. The absorbance of 100 μL aliquots of the supernatants was recorded at 405 nm using a Tecan Infinite^®^ F50 microplate reader. RBCs suspended in PBS served as the negative control, while those treated with 0.1% Triton X-100 represented the positive control. The percentage of hemolysis was determined according to Equation (2):

Hemolysis (%) *=* (*A*_sample_ − *A*_neg_)*/*(*A*_pos_ − *A*_neg_), (2)

where *A*_sample_, *A*_neg_ and *A*_pos_ refer to the absorbance values at 405 nm for the test sample, negative control and positive control, respectively.

### 2.9. Animal Model and In Vivo Photodynamic Therapy

The biological experiments were ethically reviewed and approved by the ethics committee of the First Faculty of Medicine, Charles University and by the Ministry of Education, Youth, and Sports of the Czech Republic (MSMT-2309/2018-4) in accordance with Act No. 246/1992 Sb. on the protection of animals against cruelty and Decree 419/2012 on the protection of experimental animals under the legislation of the European Parliament. The experiments were designed on the principle of the ‘Three Rs’ (replacement, reduction and refinement).

Outbred nude female mice (Hsd: athymic Nude-Fox n1nu) with a body weight ranging from 20.4 to 21.8 g were obtained from AnLab and ENVIGO (both Prague, Czech Republic) and maintained in laminar flow cabinets with radiation-sterilized SAWI bedding (Jelu-Werk; Rosenberg, Germany). The mice were provided with an irradiated Ssniff diet (Ssniff Spezialdiaeten; Soest, Germany) and unlimited access to autoclaved water. To generate the pancreatic tumor model, a mixture of 5 × 10^6^ PaTu-8902 cells with BD Matrigel matrix (VWR International; Prague, Czech Republic) were subcutaneously injected into the abdominal right flank of outbred nude mice. When the tumor volumes were ~6 mm in diameter, the mice were anesthetized with ketamine/xylazine and an aqueous dispersion of the particles (100 mL; 1.5 mg/mL) were injected intratumorally. For the PDT treatment, each PaTu tumor was irradiated after 10 min with a Quanta System IG980 excitation laser (Medicom; Prague, Czech Republic) for 3 min at 1 W (0.5 W/cm^2^; 90 J/cm^2^). The mouse weight and tumor volume were examined twice weekly, and the survival of the mice was tracked for 30 days.

### 2.10. In Vivo Optical Imaging

An optical Xtreme in vivo imager (Bruker; Ettlingen, Germany) equipped with a 980 nm laser diode (100 mW) was employed to image the treated mice with subcutaneously growing PaTu pancreatic tumor. Nu/nu female mice were scanned before and immediately after intratumoral application of the CS-UCNP@Ale-P(DMA-AEA)-PEG nanoparticles. The images were acquired in luminescence mode using a 790 nm emission band-pass filter with f/1.1 aperture and 2 × 2 pixel binning setting. Open-source FIJI ImageJ 2.9.0 was utilized for the processing of obtained images [41].

### 2.11. In Vivo MRI

CS-UCNP@Ale-P(DMA-AEA)-PEG nanoparticles were administered intratumorally and in vivo imaging was conducted before and 1 or 14 days after administration using a preclinical 1 T scanner ICON (Bruker BioSpin) equipped with a mouse body coil. The measurement protocol consisted of a localizer (fast gradient echo sequence providing three main anatomical slices for orientation) followed by a *T*_1_/*T*_2_*-weighted gradient echo in the coronal direction (TE = 4 ms, TR = 160 ms, flip angle 80°, no. of acquisitions 16, 17 slices of 1 mm thickness, field of view 28 × 56 mm^2^, matrix 128 × 256) and a similar gradient echo sequence in the axial direction (TE = 4 ms, TR = 160 ms, flip angle 80°, no. of acquisitions 32, 17 slices of 1 mm thickness, field of view 30 × 20 mm^2^, matrix 192 × 128). The sequences were positioned to cover the main part of mouse body (at least the liver, kidney and the tumor in both directions). MRI scanning was performed under general anesthesia induced and maintained by spontaneous breathing of isoflurane in air (3% for induction, 1–2% for maintenance). Vital functions were monitored during the measurement.

### 2.12. Quantification of Rare Earth Ions in Organs by ICP–MS Analysis

After PDT experiment (20 days), freshly dissected organs after intratumoral injections of the Ale-P(DMA-AEA)-PEG-coated CS-UCNPs II nanoparticles with or without conjugated VP in mice were frozen and vacuum freeze-dried for 48 h on an L4-110 PRO lyophilizer (Gregor Instruments; Sázava, Czech Republic). Concentrations of Y^3+^, Ho^3+^ and Yb^3+^ ions in powdered organs were determined by ICP–MS as described above.

## 3. Results and Discussions

### 3.1. Synthesis and Characterization of C- and CS-UCNPs

The integration of different lanthanide dopants and control of the core–shell engineering architecture enable precise modulation of upconversion luminescence and MRI contrast. Previously, we demonstrated that Fe^2+^-doped UCNPs enhanced upconversion luminescence in red-region and efficient energy transfer to a photosensitizer [34]. Here, this nanoplatform was further modified by incorporating Tm^3+^ ions to broaden the emission spectrum, thereby facilitating energy transfer to VP and multicolor bioimaging. In addition, the introduction of the Gd,Yb,Nd,Tb-doped NaHoF_4_ shell around the NaYF_4_:Yb,Er,Fe,Tm core was employed to enhance upconversion luminescence and enable bimodal optical and MR imaging [42,43]. The separation of Tm^3+^ and Nd^3+^ ions using a core–shell structure prevents cross-relaxation and a dramatic reduction in upconversion quantum yield [44,45].

**Figure 1:**
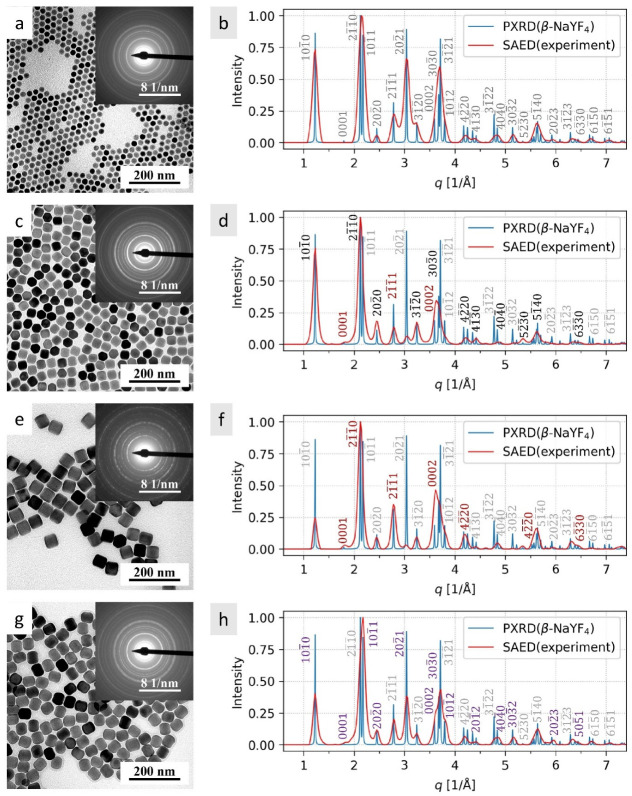
(**a**,**c**,**e**,**g**) TEM/BF micrographs and (**b**,**d**,**f**,**h**) comparison of the radially averaged TEM/SAED diffraction patterns (red lines) with the theoretically calculated PXRD patterns of hexagonal β-NaYF_4_ (blue lines). (**a**,**b**) C-UCNPs I, (**c**,**d**) C-UCNPs II, (**e**,**f**) CS-UCNPs I and (**g**,**h**) CS-UCNPs II nanoparticles. The insets in TEM/BF micrographs show the experimental TEM/SAED diffraction patterns. The Weber indices (*uvtw*) in the radially averaged diffractograms were marked with different colors corresponding to different preferred orientations of the nanocrystals: gray–general diffractions without a specific preferred orientation; black–(*hkil*) diffractions with (*l* = 0), corresponding to zone axis [*uvtw*] = [0001]; dark red–(*hkil*) diffractions with (2*h* + *k* = 0), corresponding to zone axis [*uvtw*] = [$10\bar{1}0$]; violet–(*hkil*) diffractions with (*k* = 0), corresponding to zone axis [*uvtw*] = [$11\bar{2}0$]. The insets show the corresponding experimental TEM/SAED diffraction patterns.

The successful theranostic application of UCNPs requires that the nanoparticles exhibit suitable size distribution, are biocompatible, non-toxic, colloidally stable, able to accommodate drugs and provide sufficient signal for bioimaging. The morphology, size distribution, composition, and crystal structure of the nanoparticles was thoroughly characterized by TEM (Figure 1 and Figure S1) and X-ray diffraction (Figure S2). All prepared nanoparticles (Figure 1a,c,e,g), including the CS-UCNP II ones, had a monodisperse size distribution (*Đ* < 1.01; Table 1), which ensures that the individual particles have identical physical properties to interact with biological systems. TEM/EDX spectra confirmed the expected composition of particles consisting of major elements (Na, Y, F, and Ho; Table S1), while the admixture concentrations were obtained from ICP-MS and AAS analysis (Yb, Er, Tm, Fe, Ho, Gd, and Nd; Table 2).

TEM/SAED diffraction patterns (Figure 1a,c,e,g and insets in TEM/BF micrographs) were compared with a theoretically calculated powder X-ray diffractogram (PXRD) of hexagonal NaYF_4_ crystals (Figure 1b,d,f,h). All calculations were performed with the program EDIFF as described elsewhere in [36,46]. All prepared nanoparticles exhibited the same crystal structure corresponding to hexagonal modification of sodium yttrium tetrafluoride crystals (β-NaYF_4_) as the observed peak positions corresponded to the theoretically calculated PXRD (Figure 1). The differences in peak intensities could be attributed to different preferred orientations of the nanocrystals deposited on the thin flat carbon films. The isometric, almost spherical C-UCNPs I nanoparticles (Figure 1a) were oriented randomly and, as a result, the experimental SAED intensities (Figure 1b; red line) corresponded quite well to the theoretical PXRD intensities (Figure 1b; blue line) calculated for random orientation of polycrystalline material (all diffractions in Figure 1b were marked with gray color). The bigger-faceted C-UCNPs II nanocrystals were hexagonal prisms lying mostly on their base (small hexagon projections in Figure 1c) or on their lateral face (small rectangle projections in Figure 1c). These hexagons represented the first preferred orientation with zone axis [*uvtw*] = [0001] and strong (*hkil*) diffractions with *l* = 0 (Figure 1d; black font). The second preferred orientation of C-UCNPs II was represented by the rectangles with zone axis [*uvtw*] = [$10\bar{1}0$] and strong (*hkil*) diffractions with 2*h* + *k* = 0 (Figure 1d; dark red font). The relation between crystal orientation, the corresponding zone axis [*uvtw*], and the strongest diffractions (*hkil*) are given by Weiss Zone Law as explained elsewhere [46,47]. The CS-UCNPS I nanocrystals were hexagonal prisms lying almost exclusively on their lateral faces (rectangular projections in Figure 1e), which resulted in the second type of preferred orientation observed in the previous C-UCNPS II nanocrystals (note the analogy between Figure 1d,f). The final CS-UCNPs II nanocrystals were hexagonal prisms lying on their lateral faces as well (rectangular projections in Figure 1g), but their lateral faces were formed by planes (*hkil*) = ($11\bar{2}0$) instead of planes (*hkil*) = ($10\bar{1}0$) like in the previous two cases, which resulted in yet another preferred orientation with zone axis [*uvtw*] = [$11\bar{2}0$] and strong diffractions with *k* = 0 (Figure 1g, violet font).

The crystal structure of the synthesized C and CS-UCNPs was also examined by a powder X-ray diffraction (PXRD) analysis (Figure S2). The main advantage of PXRD in comparison with TEM/SAED experiments was the higher resolution of diffraction peaks. The PXRD patterns of all particles exhibited sharp and intense peaks, indicating a well-ordered crystalline structure with a high degree of crystallinity (74 ± 2%). The diffraction positions of the CS-UCNPs II nanoparticles confirmed the presence of two phases: β-NaYF_4_ (JCPDS card no. 28-1192) in the core and β-NaHoF_4_ (JCPDS card No. 49-1896) in the shell. These two phases were isostructural, and their unit cell parameters were too close to be resolved in TEM/SAED. The hexagonal phase of CS-UCNPs II nanoparticles achieved the highest photon upconversion efficiency, highlighting their potential as NIR-to-visible nanotransducers for PDT [48].

### 3.2. Upconversion Luminescence

**Figure 2:**
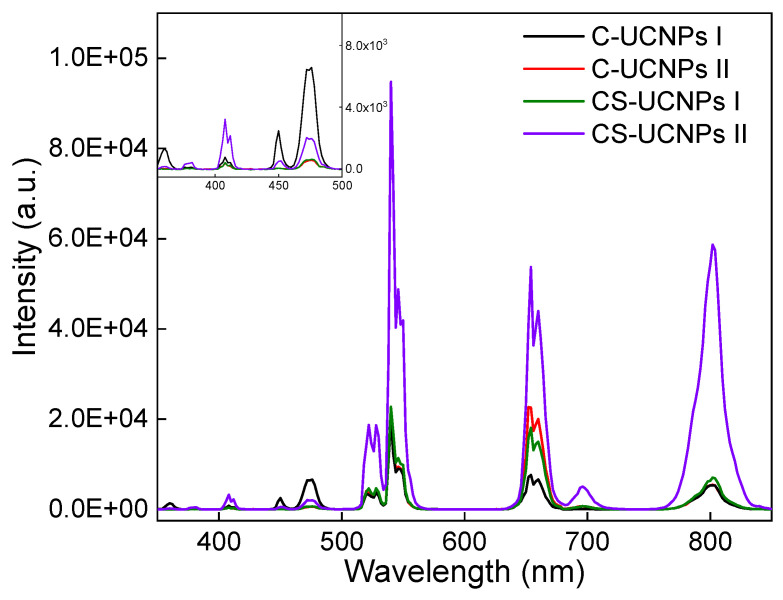
Upconversion photoluminescence spectra of C-UCNPs I, C-UCNPs II, CS-UCNPs I, and CS-UCNPs II nanoparticles (1 mg/mL) after 980 nm excitation (2.11 W/cm^2^). The inset shows magnification in the region <500 nm.

The upconversion luminescence of C- and CS-UCNPs was characterized by emission under 980 nm excitation (Figure 2). The photoluminescent spectra of C-UCNPs I exhibited characteristic peaks of Er^3+^ and Tm^3+^ in the ultraviolet, blue, green, red, and NIR regions. Their emission bands from 350 to 850 nm were ascribed to ^1^D_2_ → ^3^H_6_ (360 nm), ^1^D_2_ → ^3^F_4_ (450 nm), ^1^G_4_ → ^3^H_6_ (475 nm) and ^3^H_4_ → ^3^H_6_ (802 nm) transitions of Tm^3+^ and ^2^H_9/2_ → ^4^I_15/2_ (408 nm), ^2^H_11/2_ → ^4^I_15/2_ (520 nm), ^4^S_3/2_ → ^4^I_15/2_ (540 nm) and ^4^F_9/2_ → ^4^I_15/2_ (654 nm) transitions of Er^3+^ ions. The incorporation of Fe^2+^ ions (5 mol.%; CS-UCNPs II) into the nanoparticles quenched emissions at 360, 450 and 475 nm and increased the intensity at 650 nm by 3-fold. Moreover, the emission spectrum of C-UCNPs II nanoparticles exhibited a new peak at 696 nm ascribed to ^3^F_2,3_ → ^3^H_6_ transitions of Tm^3+^ ions. The upconversion luminescence under 980 nm excitation was slightly affected by the introduction of the undoped NaHoF_4_ shell around the C-UCNPs II nanoparticles, decreasing the emission intensity at 654 nm by 1.3-fold. This was consistent with the overlap of Ho^3+^ and Er^3+^ spectral bands with different relative intensities and energy transfer dynamics. Moreover, the CS-UCNPs I nanoparticles 1.2- or 1.4-fold increased the upconversion intensity at 520–544 and 802 nm, respectively. As expected, much more intense upconversion emission was observed for CS-UCNPs II nanoparticles, exhibiting 9-, 4-, 5-, 3-, 7- and 11-fold higher upconversion at 408–450, 475–520, 540, 654, 696, and 802 nm, respectively, compared to C-UCNPs I particles. In addition, the emission of CS-UCNPs II nanoparticles at 382 nm was detected, which is more typical for ^5^D_3_ → ^7^F_6_ transitions of Tb^3+^ ions under 980 nm excitation [43,49]. Introduction of NaHoF_4_:Gd,Yb,Nd,Tb shell on C-UCNPs II cores increased upconversion luminescence with multiple color emissions, improving energy transfer to VP and supporting bioimaging.

### 3.3. MR Relaxometry and Imaging of C- and CS-UCNPs

**Figure 3:**
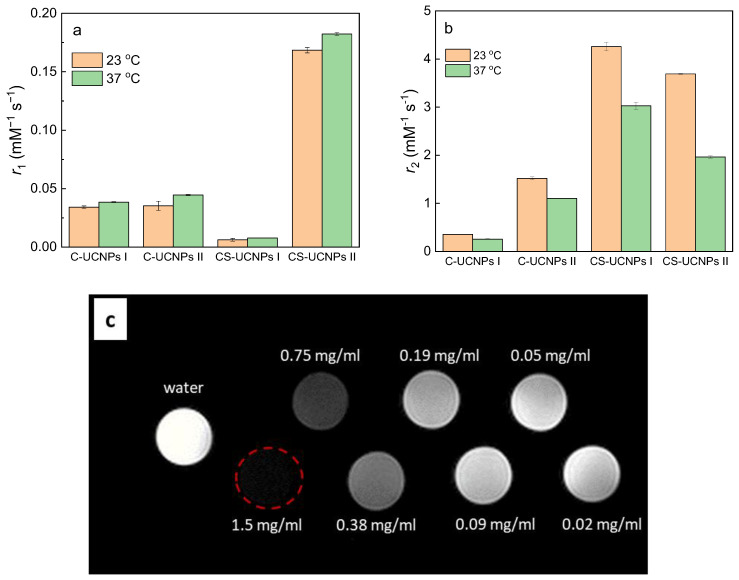
(**a**) *T*_1_ and (**b**) *T*_2_ relaxometry of C- and CS-UCNPs and (**c**) *T*_2_*-weighted MRI of phantoms containing CS-UCNPs II nanoparticles (0.02–1.5 mg/mL); acquired at (**a**,**b**) 0.5 and (**c**) 7 T. The error bar shows the standard error of the measurement.

The effectiveness of the MRI contrast agent depends on its longitudinal (*r*_1_) and transverse (*r*_2_) relaxivities, and on the *r*_2_/*r*_1_ ratio. Magnetic properties of lanthanide nanoparticles often contribute substantially to the signal and contrast in MRI due to *T*_2_* relaxation. The *r*_1_ and *r*_2_ relaxivities of aqueous dispersions of C- and CS-UCNPs were investigated in 0.5 T magnetic field at 23 and 37 °C. They were calculated from the slopes of the relaxation times as a function of lanthanide and iron concentration (Figure 3a,b and Table 3). At both temperatures, *r*_1_ relaxivities of all particles were lower than the *r*_2_ relaxivities. While *r*_1_ relaxivities of C-UCNPs I-II and CS-UCNPs I were monitored in the range of 0.01–0.05 s^−1^/mM, the incorporation of even a small amount of Gd (0.81 mM) in the shell significantly increased *r*_1_ relaxivity to 0.17 and 0.18 s^−1^/mM at 23 and 37 °C, respectively. According to the Solomon–Bloembergen–Morgan theory of relaxation, increasing number of paramagnetic Gd^3+^ cations in direct contact with water molecules enhanced *r*_1_ relaxivity [50]. The small presence of Fe^2+^ ions in C-UCNPs II 4-fold increased the *r*_2_ relaxivity at both 23 and 37 °C, compared to C-UCNPs I nanoparticles. An additional enhancement was achieved by incorporating NaHoF_4_ shell around the C-UCNPs II particles. In particular, CS-UCNPs I containing NaYF_4_:Yb,Er,Fe core and NaHoF_4_ shell exhibited the highest *r*_2_ relaxivity, reaching 4.26 and 3.03 s^−1^/mM at 23 and 37 °C, respectively. Compared to CS-UCNPs I, somewhat smaller *r*_2_ relaxivities of CS-UCNPs II nanoparticles were observed after doping Gd, Tb, Yb and Nd lanthanide ions in the NaHoF_4_ shell. The observed transverse relaxation enhancement of CS-UCNPs I and II was mainly due to the contribution from Ho^3+^ through Curie-spin relaxation mechanism [51]. The resulting low *r*_2_/*r*_1_ ratio amounting to 11 at 37 °C (Table 3) reflected increased *r*_1_ relaxation caused by a higher concentration of lanthanides, particularly of Gd^3+^ ions, which may be beneficial for in vivo MRI applications [52,53].

The performance of CS-UCNPs II nanoparticles as *T*_2_-weighted MRI contrast agent was investigated in aqueous phantoms at 7 T and was in accordance with the relaxometry results (Figure 3c). *T*_2_*-weighted images of the phantom containing suspensions of nanoparticles exhibited concentration-dependent (0.02–1.5 mg/mL) negative contrast effects. The data were also comparable to those reported previously for NaHoF_4_ UCNPs, which potentially qualifies them as *T*_2_-weighted MRI contrast agent [54]. Due to its good MRI and luminescence properties, which are suitable for direct excitation of the VP as a PDT transducer, the monodisperse CS-UCNPs II nanoparticles were considered for modification with a hydrophilic polymer coating and examined for PDT treatment.

### 3.4. Functionalization of Nanoparticles with Ale-P(DMA-AEA)-PEG and VP

The biomedical application of neat UCNPs is strictly limited by their tendency to dissolve and aggregate in various biological media [55,56]. To overcome these challenges, the surfaces of CS-UCNPs II nanoparticles were coated with the biocompatible Ale-P(DMA-AEA) polymer and subsequently PEGylated to form CS-UCNP@Ale-P(DMA-AEA)-PEG particles. This was in agreement with previously published data, where this combination of polymers increased the chemical and colloidal stability of UCNPs under physiological conditions, making the particles biocompatible and non-toxic [34]. More-over, the presence of amino groups in Ale-P(DMA-AEA)-PEG enabled further covalent conjugation of the hydrophobic VP photosensitizer, allowing the particles to act as a both imaging and therapeutic agent. This prevented aggregation and premature release of VP, thereby preserving its photochemical efficacy.

**Figure 4:**
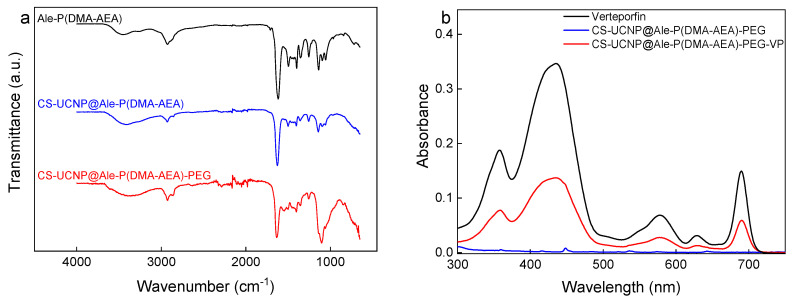
(**a**) ATR-FTIR spectra of Ale-P(DMA-AEA) and CS-UCNPs II nanoparticles coated with Ale-P(DMA-AEA) and Ale-P(DMA-AEA)-PEG. (**b**) UV–Vis spectra of pure verteporfin, CS-UCNP@Ale-P(DMA-AEA)-PEG and CS-UCNP@Ale-P(DMA-AEA)-PEG-VP nanoparticles.

The hydrodynamic diameter *D*_h_ of neat CS-UCNPs II nanoparticles in water was 257 ± 6 nm, which was much larger than their number-average size (*D*_n_ = 48 ± 3 nm; Table 1). This was associated with the aggregation of particles stabilized only by electrostatic repulsion due to a positive ζ-potential (26 ± 1 mV), which was not large enough to ensure colloidal stability [57]. After coating CS-UCNPs II nanoparticles with Ale-P(DMA-AEA), their *D*_h_ and ζ-potential decreased to 135 ± 1 nm and 13 ± 1 mV, respectively. Compared to CS-UCNPs II, the smaller size of CS-UCNP@Ale-P(DMA-AEA) nanoparticles was attributed to the effective stabilization of the particle dispersion with the polymer. The ATR-FTIR spectrum of the CS-UCNP@Ale-P(DMA-AEA) nanoparticles exhibited characteristic Ale-P(DMA-AEA) peaks at 2930 and 1628 cm^−1^ assigned to asymmetric ν_as_(CH_2_) and ν(C=O) stretching vibrations, respectively (Figure 4a) [56]. According to TGA, the amount of Ale-P(DMA-AEA) on the particle surface was 3.8 wt.% (Figure S3). After PEGylation, the *D*_h_ and ζ-potential of the CS-UCNP@Ale-P(DMA-AEA)-PEG nanoparticles changed slightly to 149 ± 3 nm and 10 ± 2 mV, respectively (Table 1). In the FTIR spectrum of CS-UCNP@Ale-P(DMA-AEA)-PEG nanoparticles, a new intense peak appeared at 1106 cm^−1^ attributed to ν_s_(-O-) symmetric stretching vibrations of PEG [46]. The amount of polymer shell in the PEGylated nanoparticles measured by TGA increased from 3.8 to 4.4 wt.% (Figure S3). According to TEM, modification of UCNPs with P(DMA-AEA) and PEG had almost no effect on particle size and size distribution (Table 1; Figure S4a,b). Hence, DLS, ATR-FTIR and TGA confirmed the successful modification of CS-UCNPs II nanoparticles with both Ale-P(DMA-AEA) and PEG.

**Figure 5:**
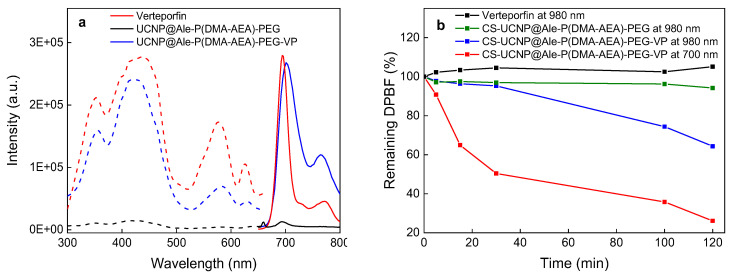
(**a**) Photoluminescence excitation (dashed; λ_em_ = 700 nm) and emission (solid; λ_ex_ = 425 nm) spectra of UCNP@Ale-P(DMA-AEA)-PEG and UCNP@Ale-P(DMA-AEA)-PEG-VP nanoparticles and (**b**) DPBF degradation at 415 nm for VP, CS-UCNP@Ale-P(DMA-AEA)-PEG and CS-UCNP@Ale-P(DMA-AEA)-PEG-VP nanoparticles in water upon 700 nm (150 W xenon lamp) and 980 nm irradiation (2.11 W/cm^2^); photodegradation (%) = 100 − remaining DPBF.

In the next step, CS-UCNP@Ale-P(DMA-AEA)-PEG particles were decorated with NHS-activated VP. Compared to particles without verteporfin, VP conjugation slightly increased *D*_h_ of CS-UCNP@Ale-P(DMA-AEA)-PEG-VP nanoparticles to 159 ± 5 nm, while ζ-potential decreased to almost zero (~1 mV; Table 1). This could have been caused by hydrophobization of the particle surface after VP conjugation, resulting in an increase in their size and the screening of surface charges. The UV–Vis spectra of CS-UCNP@Ale-P(DMA-AEA)-PEG-VP nanoparticles exhibited characteristic peaks of VP at 358, 434, 577, and 690 nm, confirming successful VP conjugation (Figure 4b). The size and morphology of CS-UCNP@P(DMA-AEA)-Ale-VP nanoparticles were similar to those of CS-UCNPs II particles (Table 1 and Figure S4c), indicating that the conjugation did not damage particles. According to UV–Vis spectroscopy, the amount of conjugated VP was 3.5 mg/g. The fluorescence spectroscopy also confirmed the presence of VP on the particle surface (Figure 5a). While CS-UCNP@Ale-P(DMA-AEA)-PEG nanoparticles did not show any typical photoluminescence excitation/emission peaks of VP, the particle conjugation with VP resulted in excitation peaks at ~423, 584 and 630 nm, which are typical of verteporfin. Moreover, the spectra of the CS-UCNP@Ale-P(DMA-AEA)-PEG-VP nanoparticles showed a shift (by 6 nm) of the emission peak maximum of VP to 701 nm, providing additional evidence of its successful conjugation to the particles. The conjugation of VP with CS-UCNP@Ale-P(DMA-AEA)-PEG nanoparticles had no impact on their upconversion luminescence (Figure S5).

### 3.5. ROS Generation

The spectral overlap between the particle upconversion emission and the absorption Soret- and Q-bands of VP in the 400–700 nm range facilitates efficient energy transfer from the particles to the VP [58]. It initiates the ROS generation, enhancing PDT efficacy and killing tumor cells. To evaluate the potential of NIR-induced PDT, the photodynamic activity of CS-UCNP@Ale-P(DMA-AEA)-PEG-VP nanoparticles was assessed using a 1,3-diphenylisobenzofuran (DPBF) probe under 980 nm irradiation for 120 min Figure 5b and Figure S6). The NIR irradiation of a solution of DPBF in ethanol/water containing VP-conjugated particles resulted in a time-dependent decrease in DPBF absorption intensity at 415 nm, corresponding to a 35% degradation of DPBF. The highest degradation (~74%) was observed for CS-UCNP@Ale-P(DMA-AEA)-PEG-VP nanoparticles under 700 nm excitation using a high-power xenon lamp, attributed to the effective singlet oxygen generation (Figure 5b and Figure S6d). As expected, the NIR irradiation of free-VP solution in ethanol did not cause any bleaching of DPBF (Figure 5b and Figure S6a). There was almost no degradation of DPBF during the 120 min irradiation of the CS-UCNP@Ale-P(DMA-AEA)-PEG nanoparticles without VP (Figure 5b and Figure S6b). This confirmed that the CS-UCNP@P(DMA-AEA)-Ale-VP nanoparticles as a nanotransducer converted deeply penetrating 980 nm NIR light into visible emission, which could be efficiently absorbed by the photosensitizers. This enabled effective energy transfer to VP, activating it and generating cytotoxic singlet oxygen from the ambient oxygen molecules.

### 3.6. In Vitro Biocompatibility of CS-UCNP@Ale-P(DMA-AEA)-PEG-VP Nanoparticles

**Figure 6:**
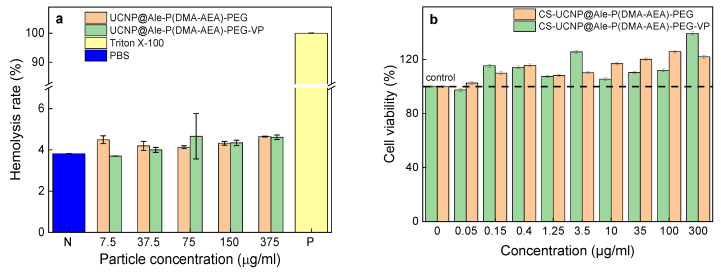
(**a**) Hemolysis rate of RBCs treated with PBS (N; negative control), Triton X-100 (P; positive control) and different concentrations of CS-UCNP@Ale-P(DMA-AEA)-PEG and CS-UCNP@Ale-P(DMA-AEA)-PEG-VP nanoparticles. (**b**) In vitro cytotoxicity of CS-UCNP@Ale-P(DMA-AEA)-PEG and CS-UCNP@Ale-P(DMA-AEA)-PEG-VP nanoparticles against human pancreatic adenocarcinoma PaTu cell line. The dashed line represents 100% viability. The error bars were calculated from three measurements.

To ensure safety and avoid side effects in both PDT and imaging, it was necessary to evaluate the toxicity and biocompatibility of CS-UCNP@Ale-P(DMA-AEA)-PEG-VP nanoparticles in the absence of 980 nm irradiation. In vitro hemolysis experiments involving CS-UCNP@Ale-P(DMA-AEA)-PEG particles with and without conjugated VP revealed no signs of negative effects (Figure 6a). The rate of hemolysis for particle concentrations up to 375 μg/mL remained similar to that observed with PBS used as a negative control, indicating that red blood cells remained unaffected. The XTT test was used to evaluate PaTu-8902 cell viability. This invasive metabolically active pancreatic ductal adenocarcinoma cell line possesses an aggressive, rapidly growing phenotype and provides a rigorous model for testing nanoparticle safety. The viability did not decrease after 48 h of incubation with both types of nanoparticles in the range of concentrations 0.05–300 μg/mL. In contrast, viability increased with the increasing particle concentration. This unexpected trend may be explained by potential interactions between the nanoparticles and the assay reagents, which could artificially enhance the metabolic readout, or by a modest stimulatory influence on cellular metabolism or survival pathways [59,60]. Consequently, CS-UCNP@Ale-P(DMA-AEA)-PEG and CS-UCNP@Ale-P(DMA-AEA)-PEG-VP nanoparticles can be considered biocompatible and non-toxic even at high concentration (0.3 mg/mL), not causing damage to red blood and pancreatic adenocarcinoma cells.

### 3.7. In Vivo NIR-Induced PDT

**Figure 7:**
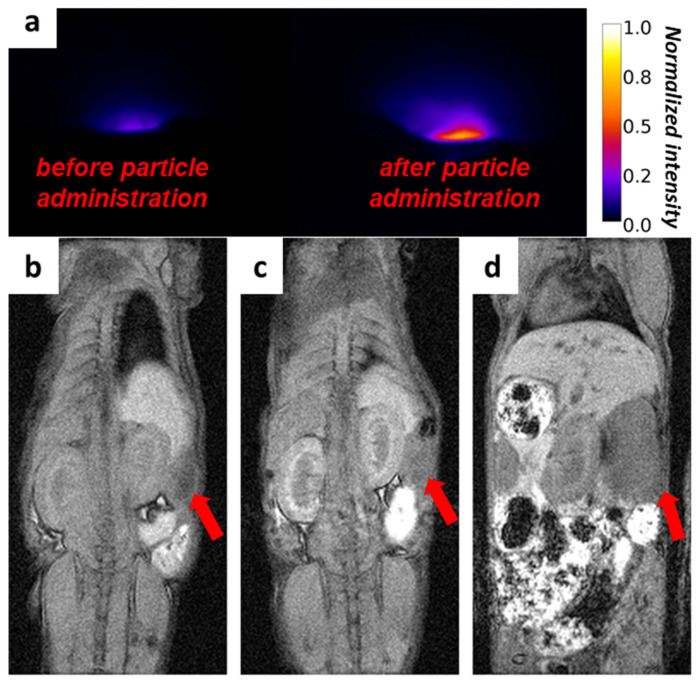
In vivo (**a**) upconversion luminescence under 980 nm excitation and (**b**–**d**) *T*_2_*-weighted MR images of the mouse bearing a subcutaneous PaTu-8902 pancreatic tumor xenograft. (**b**) Pre-injection state and (**c**) 1 and (**d**) 14 days after intratumoral administration of CS-UCNP@Ale-P(DMA-AEA)-PEG nanoparticles. Luminescence intensities were normalized to emphasize relative differences. Red arrows show the tumor area.

Finally, the potential of CS-UCNP@Ale-P(DMA-AEA)-PEG-VP nanoparticles for NIR-induced PDT was evaluated in in vivo testing on subcutaneously growing human PaTu-8902 pancreatic adenocarcinomas in nu/nu mice. Tumor-bearing mice were intratumorally injected with PBS (control group), CS-UCNP@Ale-P(DMA-AEA)-PEG and CS-UCNP@Ale-P(DMA-AEA)-PEG-VP nanoparticles and then exposed to 980 nm NIR light (0.5 W/cm^2^) for 3 min. To visualize the in vivo accumulation of particles in the tumor, mice were scanned before and after the injection of CS-UCNP@Ale-P(DMA-AEA)-PEG nanoparticles (without VP) using an Xtreme imaging system equipped with 980 nm excitation source (Figure 7a). After administration of the nanoparticles, a clear and distinct increase in the intensity of localized luminescence was observed in the tumor area, confirming the effective retention of the nanoparticles in the tumor microenvironment in vivo. MRI enabled tumor detection and monitoring of tumor growth in time (Figure 7b–d); however, it did not reveal clear evidence of the nanoparticles in the tumor. This was caused by the inability to distinguish the hypointense (negative) signal, originating from the accumulated nanoparticles, from other sources of hypointensities in cancerous environments, including necrosis, hemorrhage or various pathological changes in the tumor tissue.

**Figure 8:**
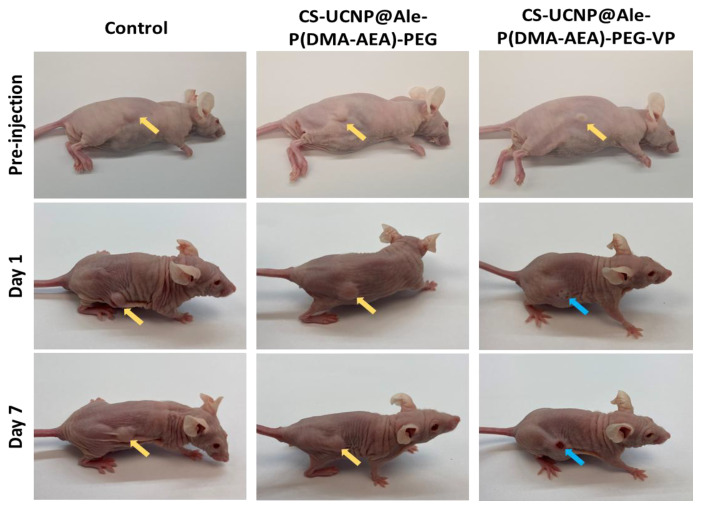
Nu/nu mice with growing PaTu human pancreatic adenocarcinoma before and 1 and 7 days after NIR-induced PDT. Mice were intratumorally injected with 100 µL of PBS (control group) or CS-UCNP@Ale-P(DMA-AEA)-PEG and CS-UCNP@Ale-P(DMA-AEA)-PEG-VP nanoparticles and irradiated with a 980 nm laser 10 min after administration. Yellow arrows—the tumor area; blue arrows—necrosis in the group treated with CS-UCNP@Ale-P(DMA-AEA)-PEG-VP nanoparticles.

**Figure 9:**
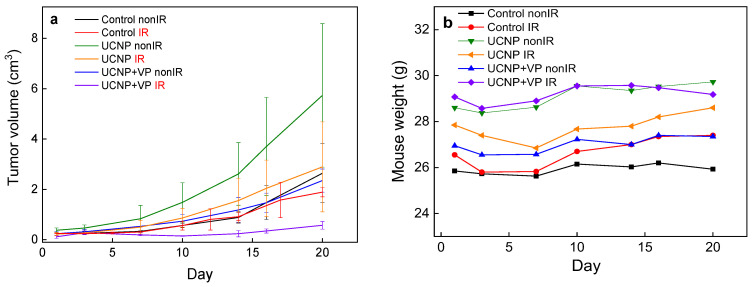
In vivo photodynamic therapy of mice with subcutaneously growing PaTu-8902 pancreatic tumor. Mice were intratumorally injected with 100 µL of PBS (control group) or CS-UCNP@Ale-P(DMA-AEA)-PEG and CS-UCNP@Ale-P(DMA-AEA)-PEG-VP nanoparticles and irradiated with a 980 nm laser 10 min after administration. Time dependence of (**a**) tumor growth and (**b**) nu/nu mice weight (*n* = 6). No evidence of systemic toxicity was documented. Control: untreated mice with intratumoral-injected PBS without irradiation. Statistical significance between CS-UCNP@Ale-P(DMA-AEA)-PEG-VP-treated group and other 6 groups—*p* < 0.05. The error bars were calculated from three measurements.

On the first day after irradiation, all mice in the CS-UCNP@Ale-P(DMA-AEA)-PEG-VP nanoparticle-treated group developed extensive necrosis, which became more pronounced on the seventh day (Figure 8). In contrast, no necrosis was observed in the non-irradiated particle-containing group, in the irradiated CS-UCNP@Ale-P(DMA-AEA)-PEG-containing group, or in the control group (PBS; Figure 8 and Figure S7). The relative tumor volumes in each treatment group increased from day 1 to day 20 after treatment (Figure 9a).

The highest relative tumor volumes were in the non-irradiated group treated with CS-UCNP@Ale-P(DMA-AEA)-PEG nanoparticles, which corroborated with previously described cell viability results. The tumor size in the control groups, i.e., in the group of mice treated with CS-UCNP@Ale-P(DMA-AEA)-PEG nanoparticles after irradiation and in the group treated with CS-UCNP@Ale-P(DMA-AEA)-PEG-VP particles without irradiation, grew in the same manner. The tumor volume at the end of the pilot study (day 20) ranged from 2 to 3 cm^3^. Specifically, statistically significant suppression of tumor growth rate was shown in the group treated with CS-UCNP@Ale-P(DMA-AEA)-PEG-VP nanoparticles after irradiation, with tumor volume ranging from 0.57 cm^3^ at day 20. Thus, the NIR-induced PDT using CS-UCNP@Ale-P(DMA-AEA)-PEG-VP nanoparticles proved its effectiveness for the treatment of pancreatic adenocarcinoma, inducing local tumor damage and exhibiting a good antitumor effect. Moreover, no significant body weight loss was observed in any of the treated groups during the observation period (Figure 9b). Tumor-bearing mice were euthanized on day 20 after treatments for quantification of rare earth ions in the organs by ICP–MS analysis. The quantification showed the accumulation of intratumorally administered CS-UCNP@Ale-P(DMA-AEA)-PEG or CS-UCNP@Ale-P(DMA-AEA)-PEG-VP nanoparticles mostly in the tumor (Table 4).

The first in vivo application of verteporfin for PDT was performed on orthotopic pancreatic cancer xenograft models using AsPC-1 and PANC-1 cell lines, which demonstrated extensive tumor necrosis dependent on the tumor aggressiveness [61]. Using VP (liposomal benzoporphyrin derivative) in the clinical study for PDT of pancreatic cancer achieved controllable tumor necrosis [62]. Nevertheless, the poor solubility of verteporfin in water, together with the need for fiber-optic irradiation with suboptimal intratumoral light deposition in the pancreas, limit the efficacy of PDT.

In recent years, some phototherapeutic agents based on VP-loaded nanoparticles have been developed for PDT with the aim of overcoming these limitations. The encapsulation of VP in nanostructured lipid carriers significantly inhibited tumor growth without visible toxicity, enhancing PDT in ovarian cancer cells [63]. VP-loaded mesoporous silica nanoparticles inhibited mouse melanoma growth, reducing the tumor mass by 50.2 ± 6.6% compared to the untreated mice [64]. A pure VP nanoformulation formed from VP dimers showed superior tumor control and prolonged animal survival in glioblastoma in vivo [40]. The incorporation of VP into poly(lactic-*co*-glycolic acid) nanoparticles functionalized with a cell-penetrating HIV transactivator of transcription peptide reported potent ROS-mediated killing and antitumor activity, which correlated with our results. At the same time, the combination of VP and upconversion nanoparticles for in vivo NIR-induced PDT has not yet been reported in the literature. This study thus demonstrates the potential of the newly designed CS-UCNP@Ale-P(DMA-AEA)-PEG-VP theranostic nanoparticles for NIR-triggered PDT of pancreatic cancer.

## 4. Conclusions

Theranostic upconversion nanoparticles, integrating both diagnostic and therapeutic functionalities, enable deep tissue penetration of NIR light with minimal collateral damage, representing a powerful platform for advancing personalized medicine. In this work, novel core–shell NaYF_4_:Yb,Er,Tm,Fe@NaHoF_4_:Yb,Nd,Gd,Tb UCNPs were constructed and evaluated as agents for *T*_2_ MRI/optical imaging and PDT treatment at NIR wavelengths. Efficient multicolor emission from these core–shell nanoparticles will support multiplexed imaging of various biomarkers, offering strong potential for advancing cancer diagnosis and therapy. Covalent binding of VP to the CS-UCNP@Ale-P(DMA-AEA)-PEG particle surface prevented aggregation and premature release of VP. The CS-UCNP@Ale-P(DMA-AEA)-PEG nanoparticles with and without VP exhibited superior biocompatibility, with excellent cell viability even at high concentrations. CS-UCNP@Ale-P(DMA-AEA)-PEG-VP nanoparticles served as an efficient NIR-to-visible nanotransducer and excitation source to activate VP, allowing efficient ROS generation under 700 and 980 nm irradiation and overcoming the limitations of clinical VP such as hydrophobicity. The obtained in vivo results of NIR-induced PDT open new avenues for the noninvasive therapy of human pancreatic cancer in the future. In addition, the combination of NIR luminescence and *T*_2_-weighted MR imaging in one nanosystem is of vital importance to meet the ever-growing performance demand of the multimodal bioprobes. This is a promising strategy for improving PDT outcomes in the treatment of deep-seated tumors.

## Figures and Tables

**Table 1:** Characterization of the nanoparticles.

Particles	*D*_n_ (nm)	*Đ*	*D*_h_ (nm)	*PD*	ζ-Potential (mV)
C-UCNPs I (NaYF_4_:Yb,Er,Tm)	20 ± 1	1.01	149 ± 3	0.15	30 ± 2
C-UCNPs II (NaYF_4_:Yb,Er,Tm,Fe)	36 ± 1	1.01	175 ± 4	0.12	32 ± 3
CS-UCNP I (NaYF_4_:Yb,Er,Tm,Fe@NaHoF_4_)	52 ± 2	1.01	265 ± 7	0.12	27 ± 2
CS-UCNP II (NaYF_4_:Yb,Er,Tm,Fe@NaHoF_4_:Gd,Yb,Nd,Tb)	47 ± 3	1.01	257 ± 6	0.10	26 ± 1
CS-UCNP@Ale-P(DMA-AEA)	47 ± 3	1.01	135 ± 1	0.17	13 ± 1
CS-UCNP@Ale-P(DMA-AEA)-PEG	47 ± 3	1.01	149 ± 3	0.17	10 ± 2
CS-UCNP@Ale-P(DMA-AEA)-PEG-VP	48 ± 4	1.02	159 ± 5	0.15	1 ± 1

C-UCNPs and CS-UCNPs–core and core–shell upconversion nanoparticles; Ale-P(DMA-AEA)–alendronate-terminated poly(*N*,*N*-dimethylacrylamide-*co*-2-aminoethyl acrylate); PEG–poly(ethylene glycol); VP–verteporfin; *D*_n_–number-average diameter (TEM), *Ð*–dispersity (TEM), *D*_h_–hydrodynamic diameter (DLS), *PD*–polydispersity (DLS).

**Table 2:** Concentrations of lanthanides and iron ions in C- and CS-UCNPs used for MR relaxometry and imaging.

Particles	[Yb^3+^] (mM)	[Er^3+^] (mM)	[Tm^3+^] (mM)	[Fe^2+^] (mM)	[Ho^3+^] (mM)	[Gd^3+^] (mM)	[Nd^3+^] (mM)	∑ (mM)
C-UCNPs I	2.8	0.03	0.1	-	-	-	-	2.9
C-UCNPs II	2.5	0.03	0.2	0.002	-	-	-	2.7
CS-UCNPs I	2.0	0.02	0.2	0.001	7.4	-	-	9.6
CS-UCNPs II	3.7	0.02	0.1	0.004	4.1	0.8	0.8	9.6

**Table 3:** Relaxometry of aqueous C- and CS-UCNP dispersions measured at 23 and 37 °C.

Particles	23 °C			37 °C		
	*r*_2_ (s^−1^/mM)	*r*_1_ (s^−1^/mM)	*r*_2_/*r*_1_ *	*r*_2_ (s^−1^/mM)	*r*_1_ (s^−1^/mM)	*r*_2_/*r*_1_ *
C-UCNPs I	0.36	0.03	10	0.26	0.04	7
C-UCNPs II	1.52	0.04	43	1.10	0.05	25
CS-UCNPs I	4.26	0.01	676	3.03	0.01	386
CS-UCNPs II	3.69	0.17	22	1.97	0.18	11

* *r*_2_*/r*_1_ was calculated from the precise relaxivity values.

**Table 4:** ICP–MS analysis of rare earth ions in mice organs 20 days after intratumoral administration of particles.

Particles	Organs	Y (mg/kg)	Ho (mg/kg)	Yb (mg/kg)
CS-UCNP@Ale-P(DMA-AEA)-PEG	Liver	0.19	0.16	0.09
	Kidneys	0.11	0.08	0.07
	Spleen	0.23	0.24	0.17
	Tumor	315.6	426.9	279.2
CS-UCNP@Ale-P(DMA-AEA)-PEG-VP	Liver	0.23	1.46	0.13
	Kidneys	0.06	0.03	0.04
	Spleen	0.15	0.10	0.08
	Tumor	823.0	910.3	660.9

## Data Availability

Data are contained within the article and Supplementary Materials.

## Supplementary Materials

The following supporting information can be downloaded at https://www.mdpi.com/article/10.3390/nano15221690/s1, Table S1: concentration of metal ions in nanoparticles, Figure S1: particle size distributions (TEM), Figure S2: experimental PXRD patterns, Figure S3: thermograms, Figure S4: TEM images, Figure S5: upconversion emission spectra, Figure S6: UV–Vis spectra, Figure S7: representative images of non-irradiated nu/nu mice.

## Abbreviations

The following abbreviations are used in this manuscript:

PDT	Photodynamic therapy
ROS	Reactive oxygen species
PS	Photosensitizer
NIR	Near-infrared
DMEM	Dulbecco’s modified Eagle medium
FBS	Fetal bovine serum
UCNPs	Upconversion nanoparticles
CS-UCNPs	Core–shell upconversion nanoparticles
P(DMA-AEA)-PEG-Ale	Alendronate-terminated poly(*N,N*-dimethylacrylamide-*co*-2-aminoethyl acrylate)-*graft*-poly(ethylene glycol)
VP	Verteporfin
MRI	Magnetic resonance imaging
NHS-PEG	Propargylacetamido poly(ethylene glycol)
