# Supplementary material for: Theranostic Verteporfin-Conjugated Upconversion Nanoparticles for Cancer Treatment
Source: Nanomaterials (Basel). 2025 Nov 7;15(22):1690. doi: 10.3390/nano15221690 (PMC12655723; doi:10.3390/nano15221690)
Supplement: Supplementary file 1 [file nanomaterials-15-01690-s001.zip › nanomaterials-3930299-supplementary.pdf]

nanomaterials

MDPI

*Supporting Information*

# Theranostic Verteporfin-Conjugated Upconversion Nanoparticles for Cancer Treatment

**Oleksandr Shapoval [1,*], Vitalii Patsula [1], David Větvička [2], Miroslav Šlouf [1], Martina Kabešová [2], Taras Vasylyshyn [1], Ludmila Maffei Svobodová [2], Magdalena Konefal [1,3], Olga Kočková [1], Jan Pankrác [4], Petr Matouš [4], Vít Herynek [4] and Daniel Horák [1,*]**

1 Institute of Macromolecular Chemistry, Czech Academy of Sciences, Heyrovského Nám. 2, 162 06 Prague, Czech Republic; patsula@imc.cas.cz (V.P.); slouf@imc.cas.cz (M.Š.); vasylyshyn@imc.cas.cz (T.V.); magdalena.konefal@amu.edu.pl (M.K.); kockova@imc.cas.cz (O.K.)
2 Institute of Biophysics and Informatics, First Faculty of Medicine, Charles University, Salmovská 1, 120 00 Prague, Czech Republic; david.vetvicka@lf1.cuni.cz (D.V.); martina.kabesova@lf1.cuni.cz (M.K.); ludmila.maffei@lf1.cuni.cz (L.M.S.)
3 NanoBioMedical Centre, Adam Mickiewicz University, Wszechnicy Piastowskiej 3, 61-614 Poznań, Poland
4 Center for Advanced Preclinical Imaging, First Faculty of Medicine, Charles University, Salmovská 3, 120 00 Prague, Czech Republic; jan.pankrac@lf1.cuni.cz (J.P.); vit.herynek@lf1.cuni.cz (V.H.); petr.matous@lf1.cuni.cz (P.M.)
* Correspondence: shapoval@imc.cas.cz (O.S.); horak@imc.cas.cz (D.H.); Tel.: +420-296-809-260 (D.H.)

**Table S1.** Concentration of metal ions (at. %) in nanoparticles determined by TEM/EDX.

| Particles | Na | Y | F | Ho |
|---|---|---|---|---|
| C-UCNPs I | 16.1 | 8.0 | 75.9 | - |
| C-UCNPs II | 10.5 | 6.6 | 82.8 | - |
| CS-UCNPs I | 9.7 | 4.1 | 81.1 | 5.1 |
| CS-UCNPs II | 14.4 | 4.3 | 78.4 | 2.9 |

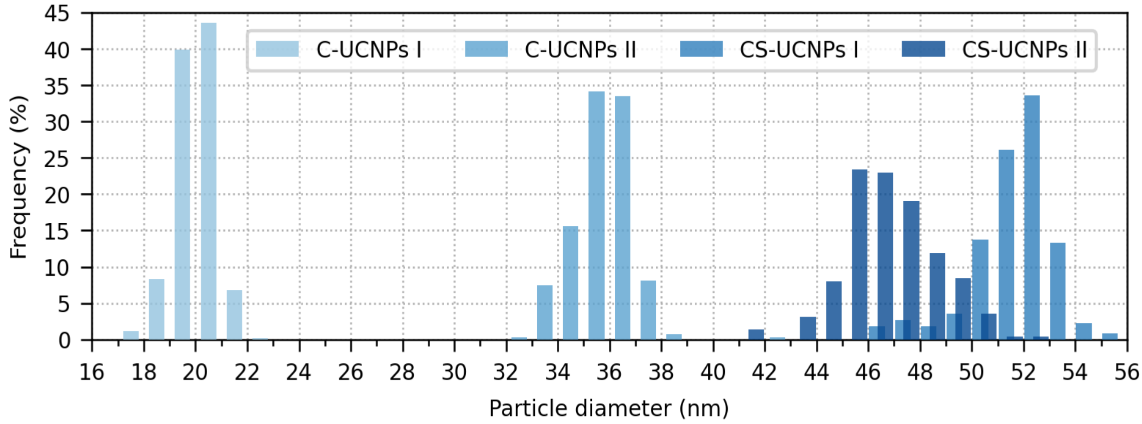


**Figure S1.** Particle size distributions obtained from the image analysis of TEM/BF micrographs for C- and CS-UCNPs.

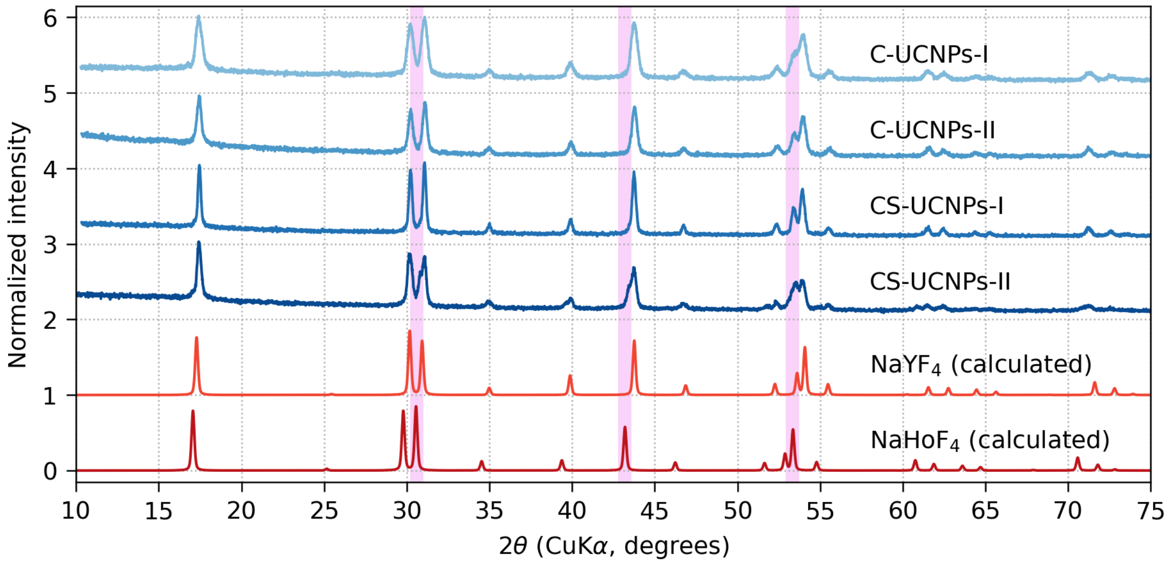


**Figure S2.** Experimental PXRD pattern of C- and CS-UCNPs nanoparticles compared to the theoretically calculated PXRD patterns of β-$NaYF_4$ (orange; JCPDS card No. 28-1192) and β-$NaHoF_4$ phase (red; JCPDS card No. 49-1896). The thin transparent vertical violet stripes indicate the strongest diffractions typical of $NaHoF_4$, which appear at different positions than the diffraction of isostructural $NaYF_4$.

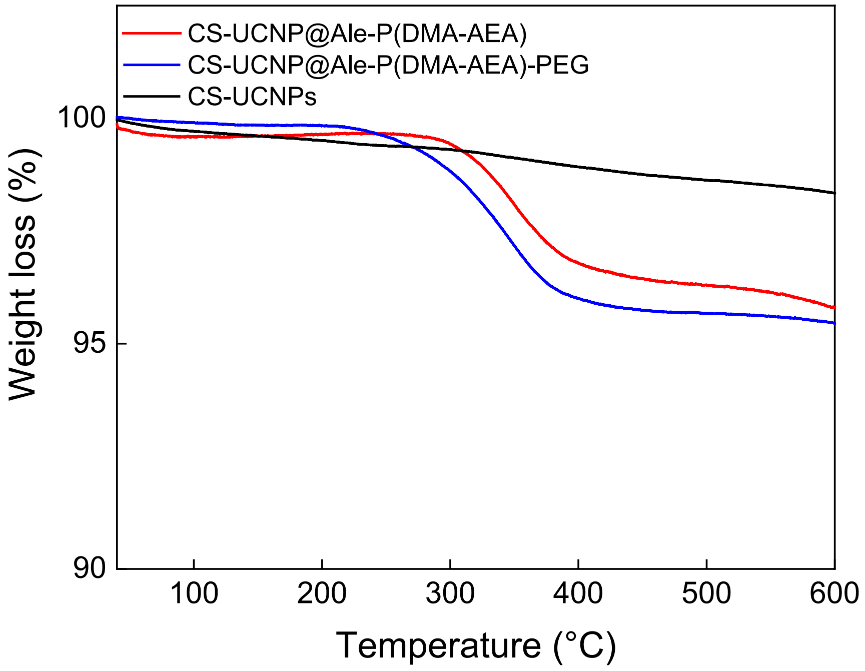


**Figure S3.** Thermograms of CS-UCNPs, CS-UCNP@Ale-P(DMA-AEA) and CS-UCNP@Ale-P(DMA-AEA)-PEG nanoparticles.

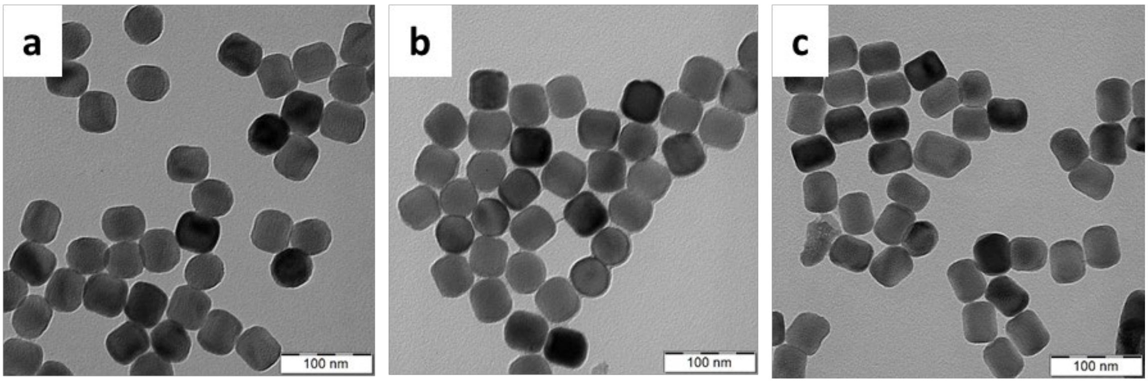


**Figure S4.** TEM images of (a) CS-UCNP@Ale-P(DMA-AEA) and (b) CS-UCNP@Ale-P(DMA-AEA)-PEG and (c) CS-UCNP@Ale-P(DMA-AEA)-PEG-VP nanoparticles.

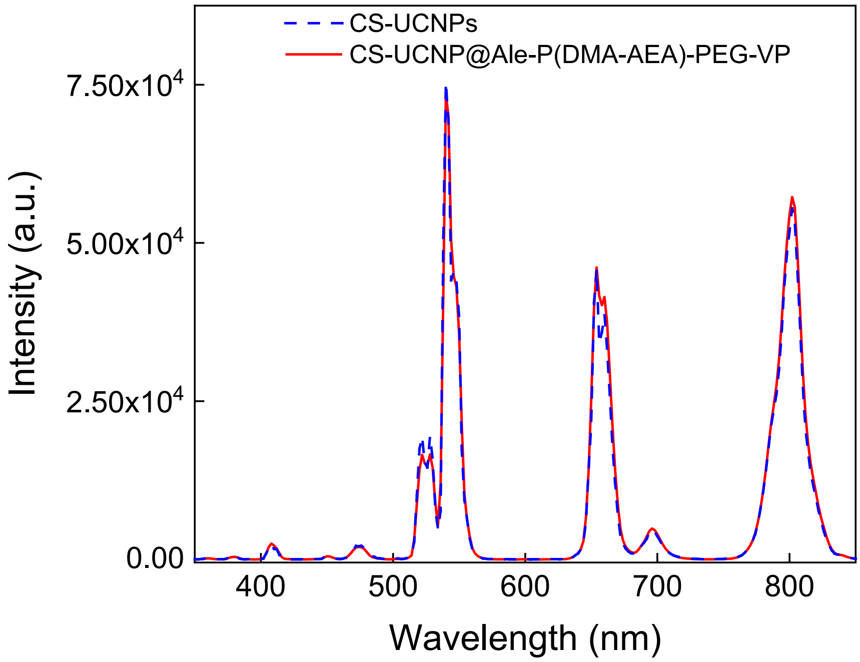


**Figure S5.** Upconversion emission spectra of CS-UCNPs II and CS-UCNP@Ale-P(DMA-AEA)-PEG-VP nanoparticles in water (1 mg/ml) excited at 980 nm with laser power densities of 2.11 W/cm².

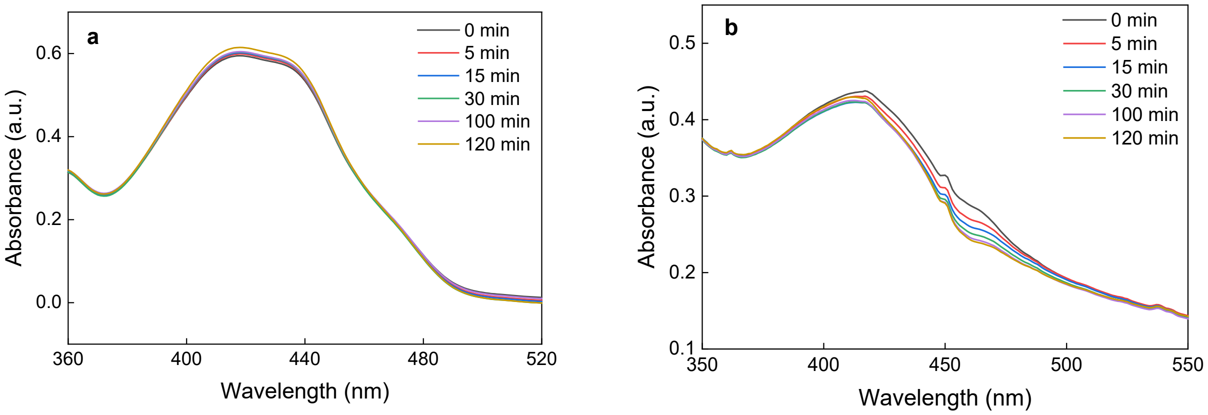

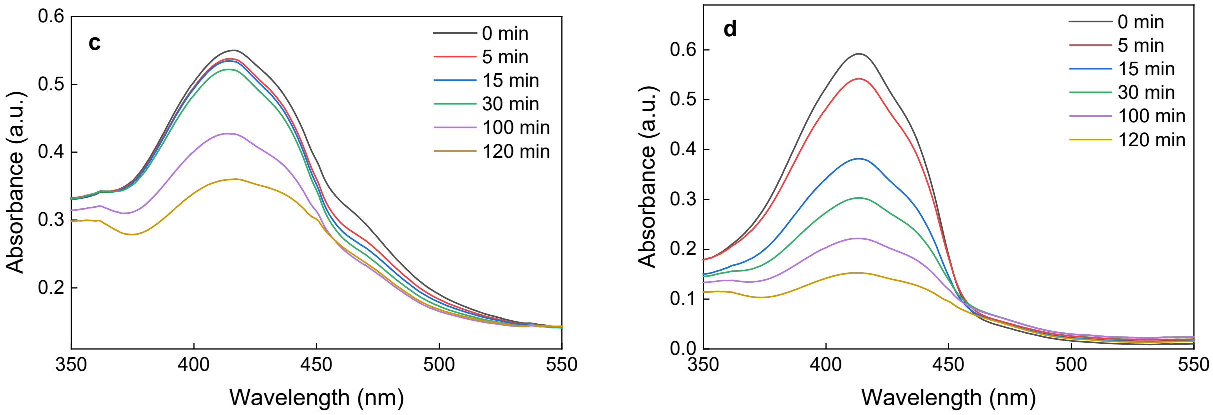


**Figure S6.** UV–Vis spectra of DPBF in ethanol/$H_2O$ mixture (50:50 v/v) containing (a) VP, (b) CS-UCNP@Ale-P(DMA-AEA)-PEG and (c, d) CS-UCNP@Ale-P(DMA-AEA)-PEG-VP nanoparticles (2 mg/ml) at different irradiation times and (a-c) 980 nm excitation (2.11 W/cm$^2$) and (d) at 700 nm excitation (150 W xenon lamp).

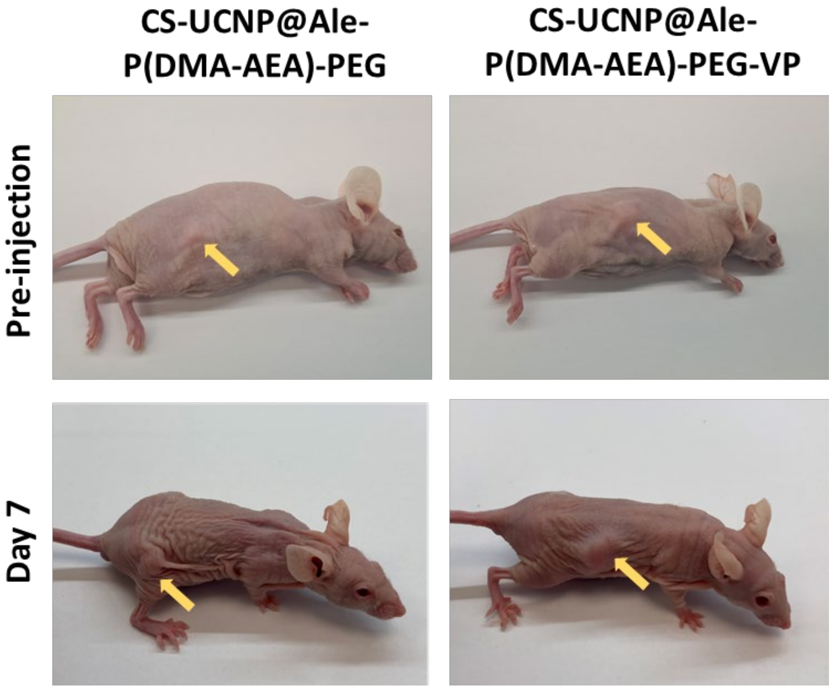


**Figure S7.** Nu/nu mice with growing PaTu human pancreatic adenocarcinoma before (upper line) and 7 days after (lower line) intratumoral administration of CS-UCNP@Ale-P(DMA-AEA)-PEG and CS-UCNP@Ale-P(DMA-AEA)-PEG-VP nanoparticles without irradiation. Yellow arrows show the tumor area.

**Disclaimer/Publisher's Note:** The statements, opinions and data contained in all publications are solely those of the individual author(s) and contributor(s) and not of MDPI and/or the editor(s). MDPI and/or the editor(s) disclaim responsibility for any injury to people or property resulting from any ideas, methods, instructions or products referred to in the content.
